# G protein‐coupled oestrogen receptor promotes cell growth of non‐small cell lung cancer cells *via* YAP1/QKI/circNOTCH1/m6A methylated NOTCH1 signalling

**DOI:** 10.1111/jcmm.15997

**Published:** 2020-11-25

**Authors:** Yi Shen, Chong Li, Lin Zhou, Jian‐An Huang

**Affiliations:** ^1^ Department of Pulmonary and Critical Care Medicine The First Affiliated Hospital of Soochow University Suzhou China; ^2^ Department of Pulmonary and Critical Care Medicine The Third Affiliated Hospital of Soochow University Changzhou China

**Keywords:** circular RNA, GPER, NOTCH1, NSCLC

## Abstract

Results from various studies reveal that the role of G protein‐coupled oestrogen receptor (GPER) is cancer‐context dependent, and the function of GPER in non–small‐cell lung cancer (NSCLC) is still unclear. The present study demonstrated that neoplasm lung tissues expressed higher level of GPER compared with the normal lung tissues. The clinical data also showed that GPER expression level was positively correlated with the tumour stage of NSCLC. Our experimental data confirmed that GPER played an oncogenic role to promote cell growth of NSCLC cells. Mechanistic dissection revealed that GPER could modulate the NOTCH1 pathway to regulate cell growth in NSCLC cells. Further exploration of the mechanism demonstrated that GPER could up‐regulate circNOTCH1, which could compete with NOTCH1 mRNA for METTL14 binding. Because of the lack of m6A modification by METTL14 on the NOTCH1 mRNA, NOTCH1 mRNA was more stable and much easier to undergo protein translation. Subsequently, we found that GPER could prevent YAP1 phosphorylation and promote YAP1‐TEAD's transcriptional regulation on QKI, a transacting RNA‐binding factor involved in circRNA biogenesis, to facilitate circNOTCH1 generation. Supportively, data from preclinical mice model with implantation of H1299 cells also demonstrated that knock‐down of circNOTCH1 could block GPER‐induced NOTCH1 to suppress NSCLC tumour growth. Together, our data showed that GPER could promote NSCLC cell growth via regulating the YAP1/QKI/circNOTCH1/m6A methylated NOTCH1 pathway, and targeting our identified molecules may be a potentially therapeutic approach to suppress NSCLC development.

## 1 INTRODUCTION

Lung cancer is the most prominent cause of global cancer‐related death. The five‐year mortality is approximately 80% due to unsatisfied early diagnosis and curative therapeutic strategy.^1^, ^2^ Non–small‐cell lung cancer (NSCLC) accounts for 85% of the total lung cancers and is pathologically subtyped into adenocarcinoma, squamous cell carcinoma (SCC) and large cell carcinoma (LCC).^3^ Owing to the improved understanding of NSCLC tumorigenesis and progression, new biomarker–targeted therapies have advanced the overall survival of NSCLC patients.^4^ Even though current therapies benefit patients a lot, the 5‐year overall survival (OS) rate of NSCLC patients in advanced stage was still less than 5%,^5^ suggesting there is urgent need to develop novel therapeutic strategies to battle against this type of disease.

The seven‐transmembrane G protein‐coupled oestrogen receptor (GPER, also known as GPR30) is one member of the G protein‐coupled receptor (GPCR) family. The discovery of GPER suggests an additional mechanism through which oestrogen (E2) could exert its effects. Increasing evidence has demonstrated that E2 exerts multiple biological effects through GPER but not the classical oestrogen receptors ERα and ERβ.^6^ G1, the first GPER‐specific agonist, which was screened out from a library of 10,000 molecules, manifested high affinity towards GPER. G15 was first identified as a GPER‐selective antagonist in 2009.^7^ Recent study has reported that GPER antagonist G15 could prevent oestrogen‐induced cancer development of NSCLC.^8^ In breast cancer, several studies have revealed that GPER could suppress YAP1 phosphorylation by turning off the Hippo pathway.^9^, ^10^ Dephosphorylated YAP1 acted as a co‐transcriptional factor through binding the transcriptional enhancer‐associated domain (TEAD) transcription factors to regulate downstream genes, which were involved in tumour initiation and progression.^11^, ^12^ However, the mechanism of how GPER regulates NSCLC cell growth is still unclear.

It has been validated that NOTCH1 can influence proliferation, apoptosis and differentiation of various cancers.^13^, ^14^ Activation of NOTCH1 signalling expands the population of cancer stem‐like cells (CSC) in breast cancer^15^ as well as ablate chemosensitivity during palliative therapy.^16^ Accumulating literature has reported that oestrogen could up‐regulate NOTCH1 to promote cancer cell proliferation.^17^, ^18^ Mechanistic dissection demonstrated that oestrogen activated oestrogen receptors (ERs) pathways to regulate NOTCH1 expression.^17^ However, Notch signalling has also been activated in triple‐negative breast cancer,^19^ implying GPER, instead of ERs, might activate the Notch pathway.

Circular RNAs (circRNAs) were first identified as by‐products of mRNA splicing in the early 1990s.^20^ Attributing to the novel sequencing technologies and bioinformatics, more and more circRNAs have been identified and proved to be functional in physiological and pathological cellular events.^21^ Encouragingly, circRNAs can be developed either as diagnostic/prognostic biomarkers or therapeutic targets in the treatments of various cancers.^22^, ^23^, ^24^ Studies displayed that circRNA can regulate gene expression as competitive endogenous RNAs (ceRNAs) or protein sponges.^25^, ^26^, ^27^, ^28^, ^29^, ^30^ Particularly, circRNA could regulate its host gene expression and was involved in tumorigenesis and tumour progression.^31^ For example, circRNA‐ENO1 up‐regulated ENO1 expression to promote lung adenocarcinoma progression.^32^ However, so far, the relationship between circNOTCH1 and NOTCH1 underlying NSCLC progression is still unclear.

CircRNAs competitively combine with RNA‐binding proteins (RBP) and decrease the binding capacity of RBP with mRNA, which determines the mRNA fate.^33^, ^34^ Among these RBPs, N6‐Methyladenosine (m6A) methyltransferase is particularly important and fashionable.^35^ Emerging studies have revealed that the methylated adenosine on RNA could decrease RNA stability through recruiting YTH N6‐methyladenosine RNA‐binding protein 2 (YTHDF2).^36^ In this study, we showed that circNOTCH1 competed with NOTCH1 for the binding of methyltransferase like 14 (METTL14), decreasing NOTCH1 mRNA m6A methylation and increasing NOTCH1 mRNA stability.

Together, our data verified that GPER exerted an oncogenic effect on NSCLC cells through regulating YAP1/QKI/circNOTCH1/m6A methylated NOTCH1 mRNA signalling.

## 2 MATERIAL AND METHODS

### 2.1 Oncomine analysis

Oncomine (www.oncomine.org), a cancer microarray database and web‐based data mining platform, aims at integrating discovery from genome‐wide expression analyses. GPER expression data were extracted from array results of 96 samples, which were conducted by Beer DG, et al.^37^ Differential analysis of GPER was conducted between lung adenocarcinoma (LUAD) and normal tissues. Meanwhile, all the tumour samples were subtyped into high tumour stage and low tumour stage. Subsequently, GPER expression differentiation was analysed between the two groups.

### 2.2 Cell culture and reagents

Two typical NSCLC cell lines A549 and H1299 were purchased from the American Type Culture Collection (ATCC, USA). Cells were maintained in Hyclone RPMI 1640 medium (HyClone; GE Healthcare Life Sciences) with 10% foetal bovine serum (FBS), L‐glutamine, and 1% penicillin and streptomycin. All cells were incubated in a humidified atmosphere of 5% CO2 at 37ºC. Cells were validated to be mycoplasma negative, as detected by PCR.

### 2.3 Lentivirus packaging and infection

PLKO.1‐shGPER, pLKO.1‐shNOTCH1, pLKO.1‐shcircNOTCH1, pLKO.1‐shQKI, pLKO.1‐shYAP1, pLKO.1‐shMETTL4, pWPI‐oeGPER, pWPI‐oeQKI and pWPI‐oeNOTCH1 were co‐transfected with packaging plasmid psPAX2 and envelope plasmid pMD2.G into HEK293T cells following lipofectamine 3000 transfection protocol, respectively. After 48h transfection, the virus supernatants were harvested through a 0.45 µm nitrocellulose filter and used immediately or frozen at −80°C for later use. The sequences were as follows: shGPER#1 targeting sequence, 5′‐ATCGGCTTTGTGGGCAACATC‐3′; and shGPER#2 targeting sequence, 5′‐ATGAGCTTCGACCGCTACATC‐3′. shNOTCH1#1 targeting sequence, 5′‐CGCTGCCTGGACAAGATCAAT‐3′; and shNOTCH1#2 targeting sequence, 5′‐CCGGGACATCACGGATCATAT‐3′; and shQKI#1 targeting sequence, 5′‐CCGAAGCTGGTTTAATCTATA‐3′; shQKI#2 targeting sequence, 5′‐CTAGCATCATAGTGCATATAA‐3′; shYAP1#1 targeting sequence, 5′‐CCCAGTTAAATGTTCACCAAT‐3′; and shYAP1#2 targeting sequence, 5′‐GCCACCAAGCTAGATAAAGAA‐3′; shMETTL14#1 targeting sequence, 5′‐GCCGTGGACGAGAAAGAAATA‐3′; shMETTL14#2 targeting sequence, 5′‐GCTAATGTTGACATTGACTTA‐3′.

### 2.4 MTT assay

Cells were cultured in phenol red‐free medium which was supplemented with charcoal‐stripped serum for 24h. After that, cells were treated with/without G1 or G15 for 24 hours. Then, cells (0.5 × 10^3^ per well) were seeded in 96‐well plates and incubated with/without G1 (CAS 881639‐98‐1, Cayman) or G15 (CAS 1161002‐05‐6, Cayman) in different termination time. Other MTT assays were conducted as the mentioned protocol above in the cells cultured with normal medium. After that, 10 µL methylthiazolyldiphenyl‐tetrazolium bromide (MTT) stock solution (5 mg/mL; Sigma‐Aldrich) was added to each well with 100 µL media for 4 hours at 37°C. The media were replaced with 100 µL dimethyl sulfoxide (DMSO), followed by incubation for 5 minutes at room temperature. The absorbance at a wavelength of 570 nm was then measured.

### 2.5 Colony formation assay

Cells were plated at 500 per well in 6‐well plates and incubated in RPMI 1640 with 10% FBS at 37°C. Two weeks later, the cells were fixed and stained with 0.1% crystal violet. The number of visible colonies was counted manually.

### 2.6 RNA extraction and quantitative real‐time PCR (qRT‐PCR) analysis

Total RNAs were extracted using Trizol reagent (Invitrogen). 1 µg of total RNA was reverse transcribed using Superscript III transcriptase (Invitrogen). Quantitative real‐time PCR (qRT‐PCR) was conducted using a Bio‐Rad CFX96 system. SYBR green was used to determine the mRNA expression level of a gene of interest. Expression levels were normalized to the GAPDH mRNA level using the 2−ΔΔCt method.

### 2.7 Western blot

Cells were washed twice with cold PBS and lysed in RIPA buffer. Proteins (25 µg) were separated on 6%‐10% SDS/PAGE gel and then transferred onto PVDF membranes (Millipore). After blocking with 5% BSA, the PVDF membranes were incubated with appropriate dilutions of primary antibodies overnight at 4°C and then HRP‐conjugated secondary antibodies for 4 hours at room temperature. Subsequently, the ECL system (Thermo Fisher Scientific) was used to visualize. The primary antibodies used for Western blot were as following, GPER (1:1000; Abcam, # ab39742), NOTCH1 (1:1000; Abcam, # ab8925), FUS (1:1000; Abcam, # ab124923), HNRNPL (1:1000; Abcam, # ab6106), QKI (1:1000; Abcam, # ab126742), ADAR (1:1000; Abcam, # ab88574), DHX9 (1:1000; EMD Millipore, MABE878), GAPDH (1:1000; Santa Cruz, #sc‐166574), YAP1 (1:1000; Santa Cruz, #sc‐376830), P‐YAP1 (1:1000; Abcam, # ab76252) and METTL14 (1:1000; Abcam, # ab 220030).

### 2.8 RNA fluorescence in situ hybridization (FISH)

FISH was performed to detect the subcellular location of circNOTCH1 using a Dig‐labelled probe (5′‐CTCCTGCAAGCTGTGGCGGG‐3′). The signals of the probes were detected by FISH Kit (K2191050, BioChain) following the manufacturer's instructions.

### 2.9 RNA immunoprecipitation (RIP)

Cells were lysed in ice‐cold lysis buffer supplemented with RNase inhibitor after different treatments. After centrifugation, 1 mL of supernatants of each sample was pre‐cleared by protein A/G beads to remove non‐specific RNA binding for 1 hour. After centrifugation, samples were then incubated with 4 µg Ago2 (Cell Signaling Technology, #2897) or 5 µg m6A antibody (Abcam, #ab208577) overnight at 4°C. The RNA/antibody complex was washed four times by RIPA buffer supplemented with RNase inhibitor, protease inhibitor cocktail. The RNA was extracted using Trizol (Invitrogen) according to the manufacturer's protocol and subjected to qRT‐PCR analysis.

### 2.10 Chromatin immunoprecipitation assay (ChIP)

Cell lysates were pre‐cleared with normal mouse IgG and protein A‐agarose. We then added 2.0 µg anti‐YAP1 antibody to the cell lysates and incubated overnight at 4°C. IgG was used as the negative control. Specific primer sets were designed to amplify a target sequence within QKI promoter, and agarose gel electrophoresis was used to identify PCR products. The specific primers were listed as follows: the forward 5′‐TCTCAGCCGCGAATTACTCT‐3′; the reverse 5′‐TCAGCTGCACCAATGAAGTC‐3′.

### 2.11 Luciferase reporter assay

The 3kb length of QKI's promoter was constructed into pGL3‐basic luciferase reporter vector. A549 cells w/wo YAP1 shRNAs were seeded in 24‐well plates, and plasmids were transfected using lipofectamine 3000 (Invitrogen). After 36 hours transfection, luciferase activity was measured by Dual‐Luciferase Assay (Promega) according to the manufacturer's manual.

### 2.12 mRNA stability assay

H1299 cells with/without circNOTCH1 shRNAs were cultured, then actinomycin D (5 µg/mL, Apexbio) was used to block de novo RNA synthesis. Subsequently, total RNA was harvested at 0, 40, 80 and 120 minutes time points and qRT‐PCR was conducted to detect the NOTCH1 mRNA.

### 2.13 In vivo studies

Thirty‐two 6 week‐old female nude mice were purchased from the National Cancer Institute (NCI) and divided into four groups (n = 8 per group) for injections of H1299 cells transfected with (a) pLKO.1 + pWPI, (b) shcircNOTCH1 + pWPI, (c) pLKO.1 + oeGPER and (d) shcircNOTCH1 + oeGPER. Then, stable H1299 cells were harvested, washed, resuspended in serum‐free media and mixed 1:1 with Matrigel. After that, cells were injected at 1 × 10^6^ into the subcutaneous tissue of the mice. The development of tumours was monitored, measuring the length and width of the tumour once a week. The mice were killed after 8 weeks. Tumours were removed for study.

### 2.14 Statistics

Experiments were repeated at least three times with triplicate data points. The continuous data were analysed by using the Mann‑Whitney U test. Results of laboratory experiments are expressed as mean ± SD. Statistical significance for in vitro or in vivo experiments was determined using the independent‑sample *t* test. *P* < .05 was considered statistically significant.

## 3 RESULT

### 3.1 GPER promoted cell growth of NSCLC cells

**FIGURE 1:**
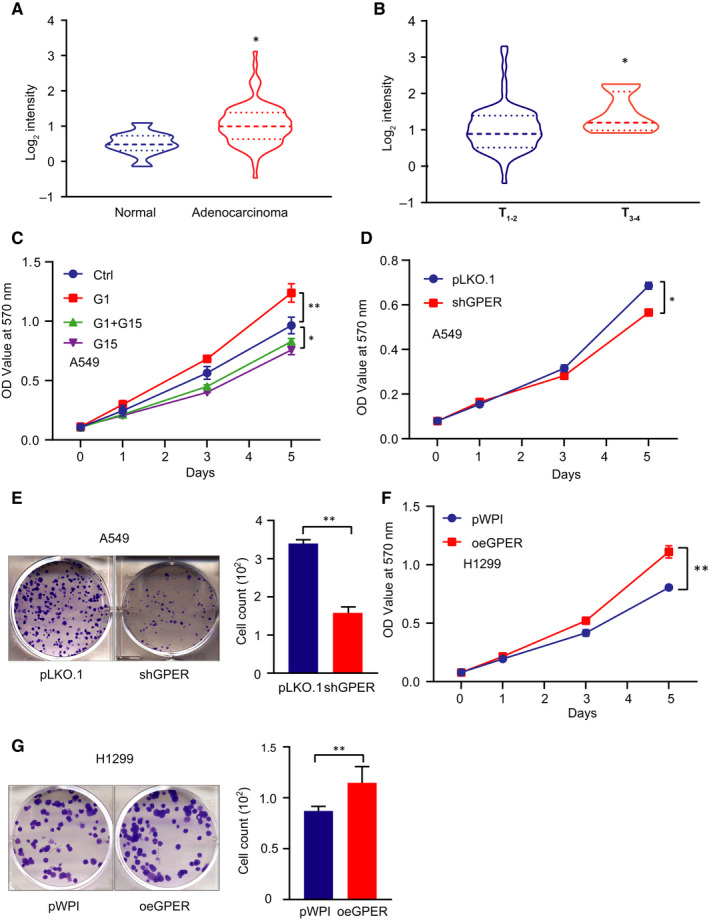
GPER promoted NSCLC cell growth. (A) GPER expression in lung cancer tissues vs normal cancerous tissues from the Oncomine data set. (B) The GPER expression level was highly expressed in T_3+4_ NSCLC samples compared with T_3+4_ ones. (C) In A549 cells pre‐treated with G1 (10 nmol/L) or G15 (1 µmol/L), MTT assay was conducted to examine cell growth. (D) MTT was used to detect cell growth of A549 cells after GPER knock‐down. (E) Colony formation assay was conducted to examine cell growth of A549 cells treated with/without shGPER. Quantification is at the right. (F) Cell growth was detected by MTT assay in H1299 cells treated with/without oeGPER. (G) Colony formation assay was conducted to examine cell growth of H1299 cells treated with/without oeGPER. Quantification was made at the right. Quantitation was presented as mean ± SD, and *P* values were calculated by *t* test, *P < .05, **P < .01

To explore the role of GPER in lung cancer, we first examined its expression level in different human lung specimens by analysing the Oncomine database of lung adenocarcinomas (www.oncomine.org). Data revealed that lung adenocarcinomas exhibited a high GPER expression profile compared with normal lung tissues (Figure 1A). The expression level of GPER was much higher in large size tumours (T_3‐4_) compared with small size ones (T_1‐2_), suggesting GPER may play an oncogenic role in the development of lung cancer (Figure 1B). To test our speculation, we treated A549 cells with GPER‐selective agonist G1 (10 nmol/L) or GPER antagonist G15 (1 µmol/L). The result of MTT assay revealed that G1 promoted cell growth while G15 suppressed cell growth of A549 cells (Figure 1C). And G1‐induced cell growth of A549 cells could be reversed by G15 treatment (Figure 1C). We selected A549 cells to conduct loss‐of‐function experiments and H1299 to perform gain‐of‐function experiments due to the relatively high level of GPER in A549 cells compared with H1299 cells (Figure S1A). First, we applied the lentivirus system to knock down GPER with shRNA (shGPER) in A549 cell line (Figure S1B) and to overexpress GPER with GPER‐CDS (oeGPER) in H1299 cell line (Figure S1C). Subsequently, MTT and colony formation assays were performed to examine cell growth of NSCLC cells. The results showed that knock‐down of GPER significantly suppressed cell growth of A549 cells (Figure 1D,E). On the contrary, overexpression of GPER dramatically promoted cell growth of H1299 cells (Figure 1F,G).

Together, the results from Figure 1A‐G and Figure S1A‐C suggested that GPER increased NSCLC cell growth.

### 3.2 GPER promoted NSCLC cell growth *via* up‐regulating the expression level of NOTCH1

**FIGURE 2:**
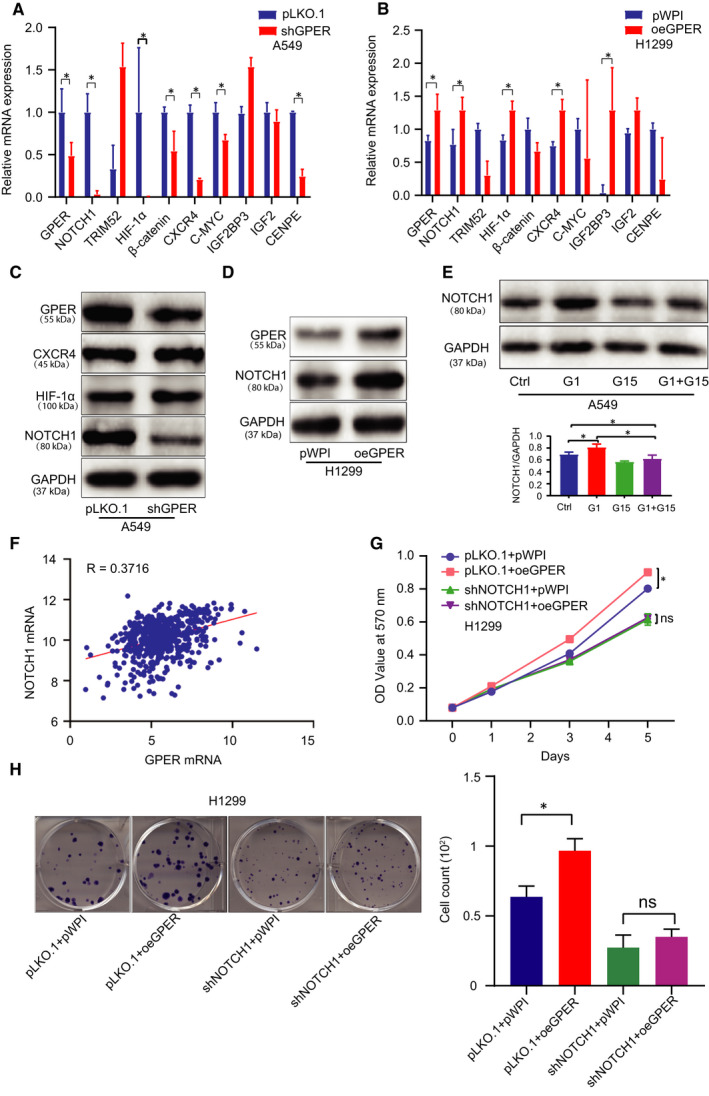
GPER promoted NSCLC growth via up‐regulating the expression level of NOTCH1. (A, B) The expression levels of cell growth/proliferation‐related oncogenes mRNA were detected by qRT‐PCR in A549 cells treated w/wo shGPER and in H1299 cells treated w/wo oeGPER. (C) The protein expression levels of the potential oncogene candidates were detected using Western blot in A549 cells after GPER knock‐down. (D) Western blot was conducted to examine NOTCH1 expression in H1299 cells after the overexpression of GPER. (E) NOTCH1 was detected in A549 cells treated with G1, G15 or G1 + G15. (F) Correlation between GPER and NOTCH1 was analysed from the TCGA data. (G) MTT assay was performed using H1299 cells transfected as indicated: pLKO.1 + pWPI, pLKO.1 + oeGPER, shNOTCH1 + pWPI, shNOTCH1 + oeGPER. (H) Colony formation assay was performed using H1299 cells transfected as indicated: pLKO.1 + pWPI, pLKO.1 + oeGPER, shNOTCH1 + pWPI, shNOTCH1 + oeGPER, and quantification was at the right. Quantitation was presented as mean ± SD, and *P* values were calculated by *t* test, *P < .05

To dissect the mechanism by which GPER altered NSCLC cell growth, we then examined the expression levels of some selective oncogenes related to cell growth/proliferation and found that knock‐down of GPER in A549 cells led to decreasing the expression levels of NOTCH1, Hif‐1α, β‐catenin, CXCR4, CENPE and C‐MYC at mRNA level (Figure 2A). In contrast, overexpression of GPER in H1299 cells led to increasing the expression of NOTCH1, Hif‐1α, IGF2BP3 and CXCR4 (Figure 2B). Western blot was conducted to detect the expression levels of three potential oncogenes in A549 cells transfected with/without (w/wo) shGPER. The result showed that only NOTCH1 protein was markedly decreased when GPER was depleted (Figure 2C). Consistently, induction of GPER increased NOTCH1 at protein level in H1299 cells (Figure 2D). To further confirm the impact on NOTCH1 expression level upon the alteration of GPER signalling, we treated A549 cells with G1, G15 and G1 + G15, respectively. The Western blotting analysis demonstrated that G1 had capacity to increase NOTCH1 level, which was blocked by G15 treatment, while G15 alone could reduce NOTCH1 expression level in A549 cells (Figure 2E). Consistently, a strongly positive correlation between GPER and NOTCH1 (R = 0.3716, *P* < .01) was observed in TCGA data set (Figure 2F). To verify that GPER‐mediated NSCLC cell growth is dependent of NOTCH1, we conducted interruption assays and the data revealed that knock‐down of NOTCH1 (Figure S1D) could partially block GPER‐induced cell growth of H1299 cells, monitored by MTT (Figure 2G) and colony formation approach (Figure 2H).

Together, the results from Figure 2A‐H and Figure S1D suggested that GPER increased NSCLC cell growth relied on NOTCH1 induction.

### 3.3 GPER functioned to positively regulate NOTCH1 expression level through circNOTCH1 in NSCLC cells

**FIGURE 3:**
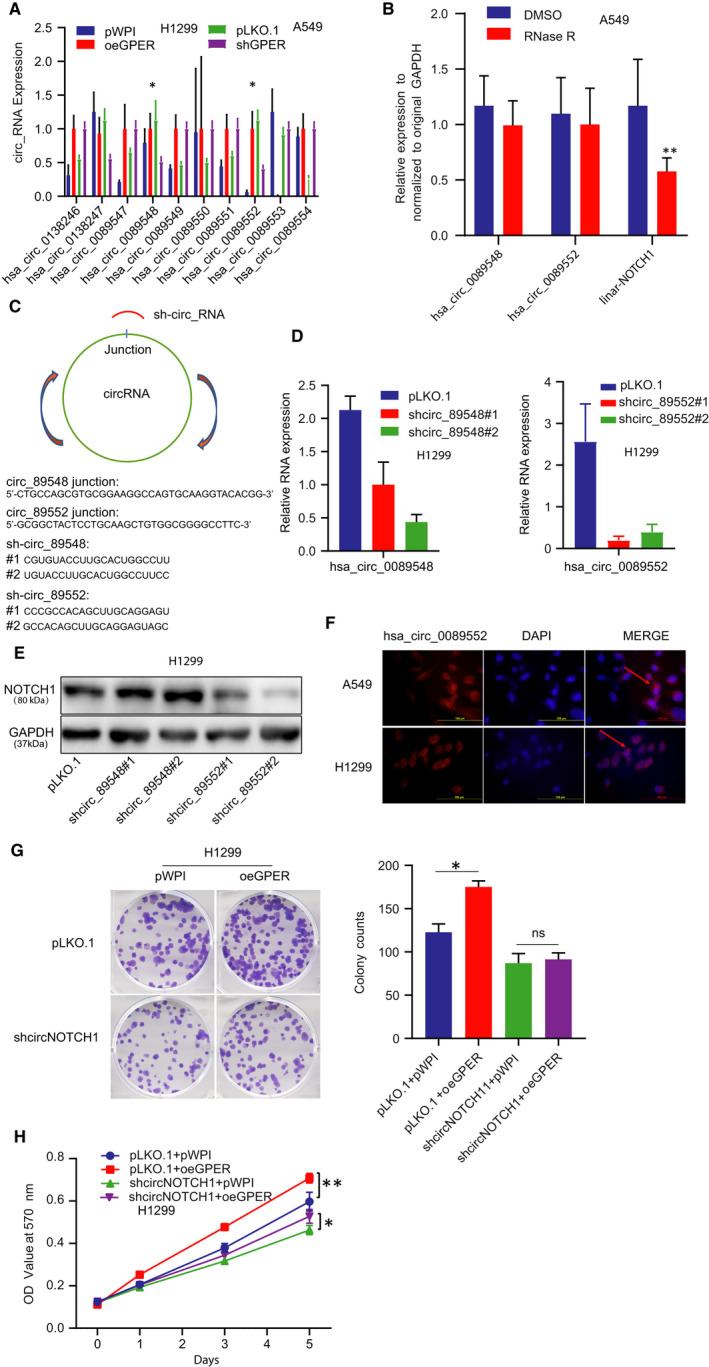
GPER functioned to positively regulate NOTCH1 expression level through circNOTCH1 in NSCLC cells. (A) We applied qRT‐qPCR to screen all the circRNAs originated from NOTCH1 gene in H1299 cells transfected with oeGPER or pWPI vector (left) and A549 cells transfected with shGPER or pLKO.1 vector (right). (B) qRT‐PCR assay was conducted to validate circRNAs expression when treated with RNase R. (C) Schematic illustration showed the principle of using shRNA to knock down the circRNAs (sh_circ_89548, sh_circ_89552). (D) qRT‐PCR assay was conducted to validate the knock‐down efficiency of circRNAs. (E) Western blot was performed to test NOTCH1 expression in H1299 cells after knocking down the two circRNAs. (F) FISH demonstrated that circNOTCH1 was predominantly localized in the cytoplasm of A549 and H1299 cells. DAPI was used to indicate the nucleus. Scale bar, 100 µm. (G) Colony formation assay was conducted to test cell growth using H1299 cells transfected as indicated: pLKO.1 + pWPI, pLKO.1 + oeGPER, shcircNOTCH1 + pWPI, shcircNOTCH1 + oeGPER, and quantification was at the right. Quantitation was presented as mean ± SD and *P* values calculated by *t* test. (H) MTT assay was conducted in H1299 cells according to the above groups. *P < .05, **P < .01

To explore the mechanism by which GPER regulated NOTCH1 expression level, we focused on the circRNAs generated from the host gene NOTCH1.^38^ We utilized the online tool circbase (http://circbase.org/) to predict all the circRNAs spliced and generated from the NOTCH1 transcript. Our detection from qRT‐PCR demonstrated that the expression levels of has_circ_0089548 and has_circ_0089552 were consistently altered upon the manipulation of GPER in A549 and H1299 cells, indicating they may be involved in the process of GPER‐induced NOTCH1 in NSCLC cells (Figure 3A). Furthermore, we conducted an exoribonuclease RNase R degrading RNA experiment to confirm the circular property of hsa_circ_0089548 and hsa_circ_0089552. The result revealed that the two circRNAs were resistant to RNase R treatment when linear‐NOTCH1 mRNA was used as a control (Figure 3B). We then applied the lentivirus system to target specific 5′‐3′ splice junctions for knocking down these two circRNAs (Figure 3C), which successfully reduced the expression levels of these two circRNAs in H1299 cells (Figure 3D). We found that reduction of has_circ_0089552 (shcirc_0089552) but not has_circ_0089548 (shcirc_0089548) could suppress cell growth of H1299 cells (Figure S1E,F). Western blotting also validated that only knock‐down of has_circ_0089552 (circNOTCH1) could decrease the protein level of NOTCH1 in H1299 cells (Figure 3E). In addition, fluorescent in situ hybridization (FISH) assay clearly demonstrated that circNOTCH1 (has_circ_0089552) was predominantly localized in cell cytoplasm of A459 and H1299 cells (Figure 3F). To further verify that circNOTCH1 was indeed involved in GPER‐mediated cell growth of NSCLC, we performed interruption assays in H1299 cells, and the results displayed that knock‐down of circNOTCH1 could block GPER‐induced cell growth of H1299 cells, monitored by colony formation assay (Figure 3G) and MTT assay (Figure 3H).

Together, the results from Figure 3A‐H and Figure S1E,F indicated that GPER functioned through circNOTCH1/NOTCH1 signalling pathway to increase NSCLC cell growth.

### 3.4 GPER regulated circNOTCH1 expression: via transcriptional regulation on QKI by YAP1/TEAD complex

**FIGURE 4:**
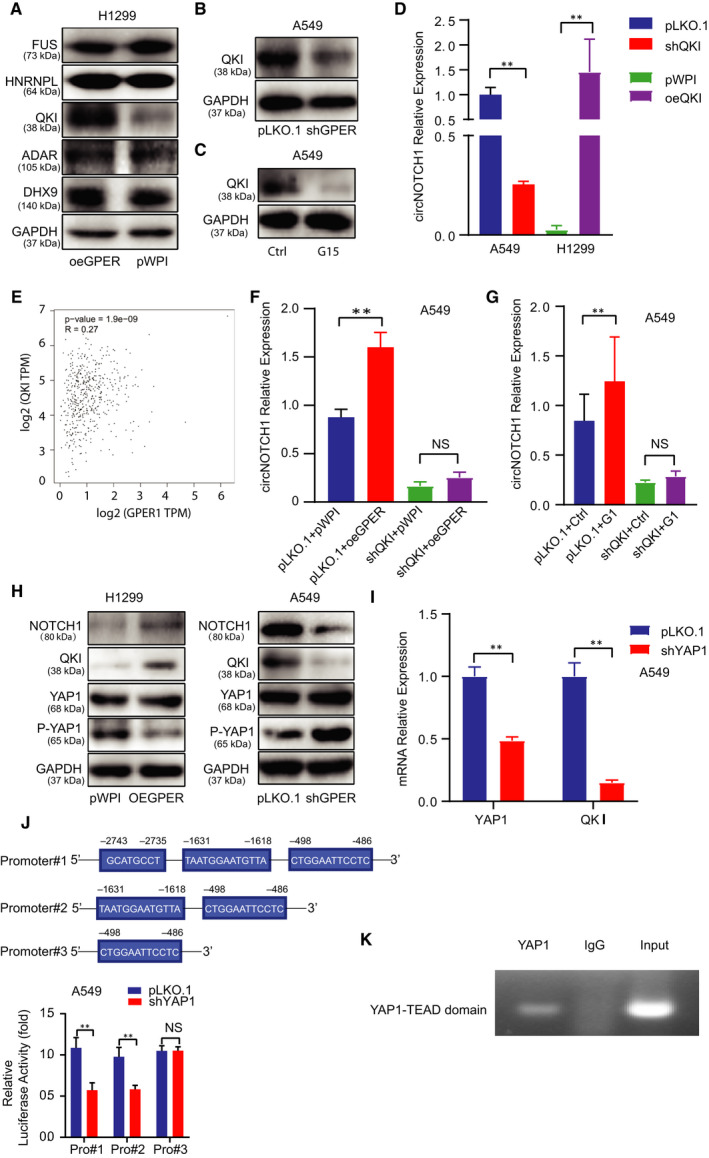
GPER regulated circNOTCH1 expression: via transcriptional regulation on QKI by YAP1/TEAD complex. (A) Western blot assay was conducted to screen a panel of transacting RNA‐binding factors in H1299 cells transfected with oeGPER or pWPI vector (right). (B) Western blot assay was performed to detect QKI expression in A549 cells w/wo shGPER. (C) Western blot assay was conducted to detect QKI expression in A549 cells treated with G15. (D) qRT‐PCR assay was conducted to detect circNOTCH1 expression in A549 cells transfected with pLKO.1 or shQKI (left) and in H1299 cells transfected with pWPI or oeQKI (right). (E) Correlation between GPER and QKI was analysed from the TCGA data. (F) qRT‐PCR assay was conducted to test circNOTCH1 expression using A549 cells transfected as indicated: pLKO.1 + pWPI, pLKO.1 + oeGPER, shQKI + pWPI, shQKI + oeGPER. (G) qRT‐PCR assay was conducted to test circNOTCH1 expression using A549 cells transfected with pLKO.1 or shQKI and subsequently treated with mock or G1. (H) Western blot assay was performed to examine NOTCH1, QKI, YAP1 and P‐YAP1 levels in H1299 cells transfected with pWPI vector or oeGPER (left) and in A549 cells transfected with pLKO.1 vector or shGPER (right). (I) qRT‐PCR assay was conducted to show the efficiency of YAP1 knock‐down and QKI mRNA expression. (J) The luciferase assay was performed to examine three YAP1‐TEAD elements viability in A549 cells transfected w/wo shYAP1. (K) ChIP assay was conducted to confirm the second TEAD element of QKI could bind with the YAP1. Quantitation was presented as mean ± SD, and *P* values were calculated by *t* test, **P < .01

To dissect the mechanism by which GPER regulated circNOTCH1, we first checked several transacting RNA‐binding factors including ADAR, quaking (QKI), FUS, HNRNPL and DHX9, which were involved in circRNA biogenesis. Our data showed that only QKI expression level was increased upon GPER induction in H1299 cells (Figure 4A). Consistently, inhibition of GPER with shRNA (Figure 4B) or its antagonist G15 (Figure 4C) all led to decreased QKI expression in A549 cells. We then applied the lentivirus system to attenuate QKI expression level with shRNA‐QKI (shQKI #1 and shQKI #2) in A549 cells and to overexpress QKI with QKI‐CDS in H1299 cells (Figure S2A). We noticed that overexpression of QKI could increase circNOTCH1 expression in H1299 cells and knock‐down of QKI had ability to decrease circNOTCH1 expression in A549 cells (Figure 4D). Of note, we utilized the online tool (http://gepia.cancer‐pku.cn) to analyse LUAD data of The Cancer Genome Atlas (TCGA), and we observed a positive correlation between GPER and QKI, which supported our study (Figure 4E). Next, the interruption experiment was conducted to examine whether GPER functioned through QKI to regulate circNOTCH1 expression level. The result of qRT‐PCR demonstrated that knock‐down of QKI could block GPER‐induced circNOTCH1 expression level in A549 cells (Figure 4F). Similar result was gained in the shQKI A549 cells treated with G1 (Figure 4G). Recent reports illustrated that GPER could function through the Gαq‐11, PLCβ/PKC, and Rho/ROCK signalling pathways to promote YAP1 dephosphorylation, which entered the nucleus and regulated its downstream genes through the recruitment of TEADs.^9^, ^10^, ^39^ To test this mechanism in NCLSC cells, we conducted Western blot to examine the phosphorylation level of YAP1. As expected, overexpression of GPER could decrease YAP1 phosphorylation level, which was accompanied by increased expression levels of QKI and NOTCH1 in H1299 cells (Figure 4H left). On the contrary, GPER reduction by shRNA could increase the phosphorylation level of YAP1 as well as decreasing QKI and NOTCH1 in A549 cells (Figure 4H right). These results were consistent with previous study showing GPER could regulate YAP1 activation.^9^ Next, we applied the lentivirus system to knock down YAP1 in A549 cells and conducted Western blot to detect YAP1 and QKI expression. The results demonstrated that knock‐down of YAP1 suppressed QKI expression in A549 cells (Figure S2B and Figure 4I). Zanconato et al (2015) analysed ChIPseq data and demonstrated YAP‐TEAD‐driven gene signature directly contributes to breast cancer cells growth. Yu et al (2018) showed TEAD mediated the NSCLC aggressiveness induced by YAP1. Numerous studies of knowledge have illustrated that YAP1 activates the TEAD transcription factor family to output its important roles in various biological processes and human diseases.^40^, ^41^, ^42^ Here, we hypothesized that YAP1 acted as a co‐transcriptional factor combing with TEAD to regulate QKI transcription. To test our hypothesis, we used the Ensemble website with JASPAR database to screen the potential TEAD elements on the upstream 3 kb region of QKI gene locus. We found three putative TEAD elements located within the QKI's promoter region (Figure 4J upper). Then, we conducted the QKI‐based luciferase reporters by inserting different QKI's promoter regions containing different TEAD elements into the pGL3 luciferase backbone to test which TEAD element of QKI's promoter was responsible for QKI regulation by YAP1. The result demonstrated that the depletion of the second YAP1‐TEAD element blocked YAP1‐mediated transcriptional activation of QKI (Figure 4J lower). The chromatin immunoprecipitation assay (ChIP) in vivo binding assay also confirmed that YAP1‐TEAD could bind to the second TEAD element (Figure 4K).

Together, the results from Figure 4A‐K and Figure S2A,B suggested that GPER could increase circNOTCH1 expression *via* YAP1‐TEAD/QKI signalling.

### 3.5 CircNOTCH1 competitively bond with METTL14 for protecting NOTCH1 mRNA

**FIGURE 5:**
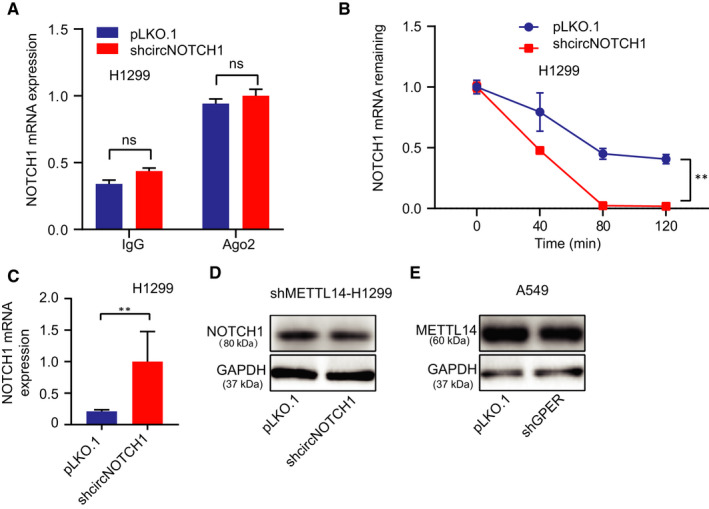
CircNOTCH1 competitively bond with METTL14 for protecting NOTCH1 mRNA. (A) qRT‐PCR assay was conducted to examine the NOTCH1 mRNA level from the anti‐Ago2 complex in H1299 cells treated w/wo shcircNOTCH1. (B) After using actinomycin D treated H1299 cells pre‐transfected w/wo shcircNOTCH1, qRT‐PCR assay was conducted to detect the NOTCH1 mRNA level at a different time (0, 40, 80, and 120 min). (C) After m6A antibody pull‐down of H1299 cells transfected w/wo shcircNOTCH1, qRT‐PCR assay was conducted to detect the NOTCH1 mRNA level. (D) METTL14‐depleted H1299 cells were transfected w/wo shcircNOTCH1, and then, Western blot assay was performed to examine the NOTCH1 protein level. (E) The METTL14 protein level was detected in A549 cells transfected w/wo shGPER by Western blot. Quantitation was presented as mean ± SD, and *P* values were calculated by *t* test, *P < .01

To determine how circNOTCH1 regulated NOTCH1 expression, we referred to the competing endogenous RNAs (ceRNAs). To test this assumption, we examined the potential regulation of NOTCH1 by miRNAs through detecting the NOTCH1 mRNA in the Argonaute 2 (Ago 2) complex using RNA interaction‐precipitation (RIP) assay (Figure S2C), because numeral studies reported that Ago 2 protein was involved in the miRNA mediated post‐transcriptional regulation on mRNA through RNA‐induced silencing complex (RISC). Unexpectedly, our data negated the assumption that circNOTCH1 regulated NOTCH1 translation through miRNAs induced post‐transcription regulation (Figure 5A). Then, we switched to mRNA stability as several studies demonstrated that circRNAs could function as protein sponges or decoys to determine mRNA fates. We conducted an mRNA degradation experiment to detect NOTCH1 mRNA stability in H1299 cells. The NOTCH1 mRNA is more much stable in the control group than that in the shcircNOTCH1 group (Figure 5B). Given the fact that N6‐Methyladenosine (m6A), the most prevalent internal modification associated with eukaryotic mRNA metabolism, plays an important role in mRNA stability,^43^ we sought to investigate whether m6A modification was involved in circNOTCH1 regulated NOTCH1 stability. Result from RNA interaction‐precipitation (RIP) using m6A antibody (Figure S2D) showed that NOTCH1 mRNA level was much higher in shcircNOTCH1 group than that in the control group (Figure 5C), suggesting a less m6A modification on NOTCH1 mRNA upon the induction of circNOTHC1 by GPER. These results implied that m6A modification on NOTCH1 mRNA decreased its stability and circNOTCH1 competitively bond with endogenous modulated m6A modification RNA‐binding protein (RBP) and released NOTCH1 mRNA. Then, we applied the online tool (starbase, http://starbase.sysu.edu.cn/degradomeRNA.php?source=ncRNA) to predict which m6A methyltransferases could combine with both NOTCH1 mRNA and circNOTCH1 and got a potential candidate, METTL14. To test whether METTL14 was involved in NOTCH1 mRNA m6A modification, we first applied the lentivirus system to knock down METTL14 in H1299 cells (Figure S2E). Then, we treated the METTL14‐depleted H1299 cells w/wo shcircNOTCH1 and examined NOTCH1 expression by Western blot. The result showed that circNOTCH1 failed to alter NOTCH1 protein level in the METTL14‐depleted H1299 cells (Figure 5D). Of note, we also noticed that GPER had little ability to regulate METTL14 expression level in A549 cells (Figure 5E), implying the regulatory specificity of circNOTCH1 towards NOTCH1.

Together, the results from Figure 5A‐E and Figure 2C‐E suggested that circNOTCH1 could increase NOTCH1 expression *via* competitively binding with the m6A methyltransferase, METTL14.

### 3.6 CircNOTCH1 depletion could block GPER‐induced tumour growth in the subcutaneous xenograft mouse model

**FIGURE 6:**
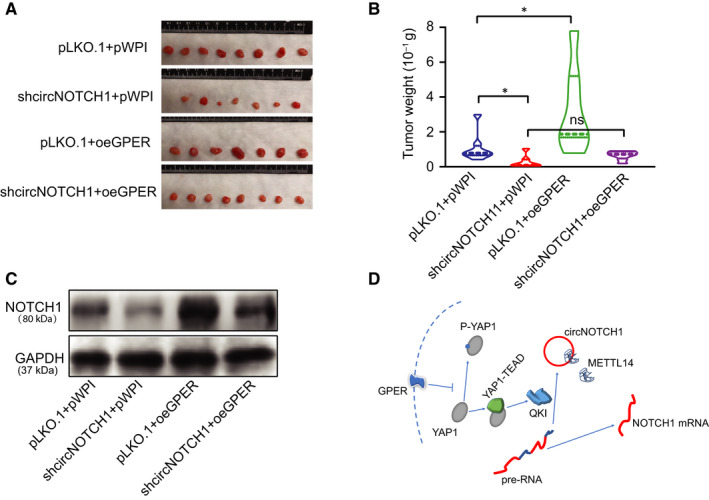
CircNOTCH1 depletion could block GPER‐induced tumour growth in the subcutaneous xenograft mouse model. (A) Subcutaneous xenograft nude mouse model was established with injections of H1299 cells transfected as indicated: pLKO.1 + pWPI, pLKO.1 + oeGPER, shcircNOTCH1 + pWPI, shcircNOTCH1 + oeGPER. Images of tumours are shown after mice were killed. (B) Quantification of tumour weights. (C) Four tumour samples were randomly picked up from each group, and NOTCH1 was detected by WB. (D) Schematic model of modulating YAP1/QKI/circNOTCH1/m6A methylated NOTCH1 signalling by GPER in non–small‐cell lung cancer. Quantitation was presented as mean ± SD, and *P* values were calculated by *t* test, *P < .05

To confirm all the above in vitro cell lines data in the vivo nude mouse model, we randomly divided mice into four groups for the subcutaneous injections of H1299 cells with pLKO.1 + pWPI (Group 1), shcircNOTCH1 + pWPI (Group 2), pLKO.1 + oeGPER (Group 3) and shcircNOTCH1 + oeGPER (Group 4). After 8 weeks, we then killed the mice and measured the size and weight of each tumour (Figure 6A,B). The results showed that mice injected with cells with pLKO.1 + oeGPER had increased tumour growth compared with the control cohorts (Figure 6A,B). However, this increased tumour growth by GPER was suppressed when circNOTCH1 was reduced (Figure 6A,B). Importantly, Western blotting analysis on tumour samples also revealed that depletion of circNOTCH1 could block GPER‐induced NOTCH1 expression (Figure 6C).

Together, results from the in vivo studies in Figure 6A‐C confirmed our in vitro studies and demonstrated that GPER could promote NSCLC progression by the modulation of YAP1‐TEAD/QKI/circNOTCH1/m6A methylated NOTCH1 signalling (Figure 6D).

## 4 DISCUSSION

Previous global statistics showed that lung cancer was the most commonly diagnosed cancer and the leading cause of cancer‐related deaths.^44^ Several in vitro and in vivo studies have proved that oestrogen could promote lung cancer proliferation.^45^ However, the therapeutic effect of pure antiestrogen (tamoxifen) was still unsatisfied. The utility of tamoxifen combined with GPER antagonists exhibited marked effects in blocking the progression of the primary breast tumour in the experimental animal model.^46^ Mechanistic study revealed that tamoxifen acted as a GPER agonist to activate GPER, which in turn provided survival signal for breast cancer cells. Consistent with this report, our study also exhibited that GPER can function through YAP1‐TEAD/QKI/circNOTCH1 signalling to regulate NOTCH1 expression and to promote NSCLC tumour growth, supplementing the oncogenic role of GPER in lung cancer development.

Among several genes related to cell growth/proliferation, NOTCH1 was proved as the downstream gene regulated by GPER. In fact, the tumour‐promoting role of NOTCH1 in NSCLC development has been well documented, and NOTCH1 inhibitors have been tested in preclinical studies of lung cancer. Furthermore, the xenografted mice model injected with H1299 cells confirmed that GPER functioned through circNOTCH1 to regulate NOTCH1 expression and lung cancer tumour growth. However, our study still showed some flaws in creating a mice model to demonstrate the therapeutic effect of GPER antagonist G15 on lung cancer progression.

Circular RNAs have been regarded as potential molecular markers for tumour diagnosis and treatment, playing an important role in tumorigenesis and progression.^47^ Numerous studies have demonstrated that circRNAs can be distinct from linear RNAs from the same host genes, and some of these circRNAs can modulate host gene expression through competing for the binding of microRNAs, RNA‐binding proteins or translation initiation complex.^31^, ^48^, ^49^, ^50^ In our study, we demonstrated that GPER could regulate NOTCH1 expression through enhancing circNOTCH1 expression, which bond with METTL14 and released NOTCH1 mRNA. METTL14, one of the m6A 'writers', increases the RNA m6A modifications influences the activity and stability of cellular mRNAs.^51^ However, the m6A modification RNAs' fate is determined by a group of RNA‐binding proteins that specifically recognize the methylated adenosine on RNA named m6A ‘readers’.^52^ For example, in breast cancer cells, METTL14 promotes cancer cell invasion and migration, but in colorectal cancer, METTL14 inhibits proliferation and metastasis.^51^, ^53^ In the present study, m6A antibody pull‐down data confirmed that knock‐down of circNOTCH1 increased the m6A modification on Nothch1 mRNA (Figure 5C). However, circNOTCH1 failed to alter NOTCH1 expression when METTL14 was depleted, and knock‐down GPER did not modulate METTL14 expression (Figure 5D,E). Combined with the RNA degradation data (Figure 5B), our data demonstrated that circNOTCH1 modulated NOTCH1 expression through competing for the binding of METTL14.

YAP1 has been regarded as an oncogene in tumorigenesis and progression through YAP1‐TEAD transcriptional regulation.^54^, ^55^, ^56^ Mechanistic dissection revealed that GPER functioned through YAP1‐TEAD to regulate QKI expression, which in turn promoted the formation of circNOTCH1 by binding to the normal linear pre‐NOTCH1.^57^ Indeed, knock‐down of QKI in A549 cells could block GPER‐induced circNOTCH1 expression and YAP1 inhibition by shRNA also significantly decreased QKI mRNA level. The luciferase reporter assay demonstrated that the second YAP‐TEAD element on the promoter of QKI was responsible for the transcriptional regulation of YAP on QKI. Together, all these data illustrated that GPER regulates circNOTCH1 expression through dephosphorylating YAP1 that promotes QKI expression.

Collectively, the findings of the present study demonstrate that GPER may promote NSCLC cell growth by regulating YAP1‐TEAD/QKI/circNOTCH1/m6A methylated NOTCH1 signalling (Figure 6D) and targeting this signalling with small molecules may be promising therapeutic strategies to retard NSCLC progression.

## CONFLICT OF INTEREST

This study has no potential conflict of interest to declare.

## AUTHOR CONTRIBUTIONS

**Yi Shen:** Conceptualization (equal); Data curation (equal); Methodology (equal); Resources (equal); Software (equal); Supervision (equal); Validation (equal); Visualization (equal); Writing‐original draft (equal); Writing‐review & editing (equal). **Chong Li:** Data curation (equal); Project administration (equal); Resources (equal); Software (equal). **Lin Zhou:** Data curation (equal); Writing‐original draft (equal); Writing‐review & editing (equal). **Jian‐An Huang:** Conceptualization (equal); Data curation (equal); Formal analysis (equal); Funding acquisition (equal); Project administration (equal); Resources (equal); Writing‐original draft (equal); Writing‐review & editing (equal).

## Supporting information

Fig S1

Fig S2

## Data Availability

The data sets used and/or analysed during the current study are available from the corresponding author on reasonable request.
