# Supplementary figures and images for: G protein‐coupled oestrogen receptor promotes cell growth of non‐small cell lung cancer cells via YAP1/QKI/circNOTCH1/m6A methylated NOTCH1 signalling
Source: J Cell Mol Med. 2020 Nov 25;25(1):284–96. doi: 10.1111/jcmm.15997 (PMC7810948; doi:10.1111/jcmm.15997)

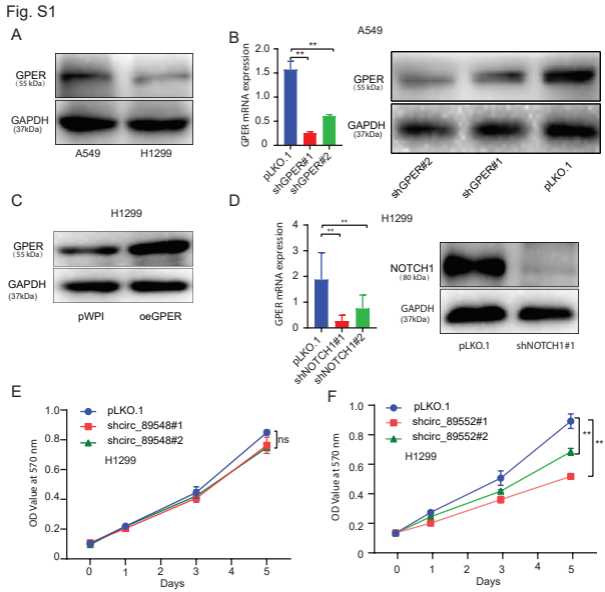

Supplement: Supplementary file 1 — Fig S1 [file JCMM-25-284-s001.pdf]

Fig. S2

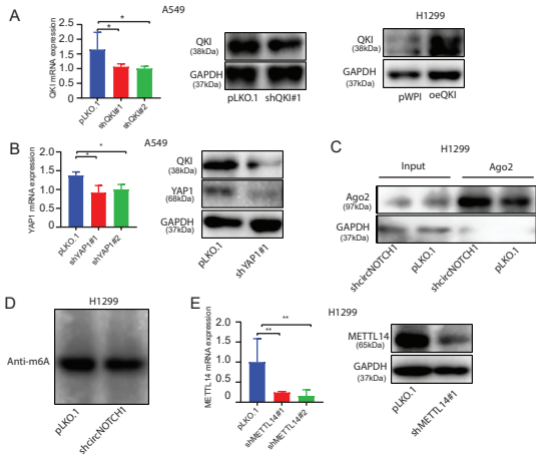

Supplement: Supplementary file 2 — Fig S2 [file JCMM-25-284-s002.pdf]
